# Association between colorectal cancer and expression levels of miR-21, miR-17-5P, miR-155 genes and the presence of *Fusobacterium nucleatum* in biopsy samples obtained from Iranian patients

**DOI:** 10.1186/s13027-023-00494-y

**Published:** 2023-03-01

**Authors:** Nazila Bostanshirin, Bahareh Hajikhani, Amir Abbas Vaezi, Fatemeh Kermanian, Fatemeh Sameni, Somayeh Yaslianifard, Mehdi Goudarzi, Masoud Dadashi

**Affiliations:** 1 Department of Microbiology, School of Medicine, Alborz University of Medical Sciences, Karaj, Iran; 2 Department of Microbiology, School of Medicine, Shahid Beheshti University of Medical Sciences, Tehran, Iran; 3 Department of Internal Medicine, Alborz University of Medical Sciences, Karaj, Iran; 4 Department of Anatomy, School of Medicine, Alborz University of Medical Sciences, Karaj, Iran; 5 Department of Microbiology, Faculty of Medicine, Shahed University, Tehran, Iran; 6 Non-Communicable Diseases Research Center, Alborz University of Medical Sciences, Karaj, Iran

**Keywords:** CRC, miR-17-5P, miR-21, miR-155, *Fusobacterium nucleatum*

## Abstract

**Background:**

Colorectal cancer (CRC) is considered the second-deadliest and third-most common malignancy worldwide. Studying the carcinogenic mechanism of bacteria or their role in aggravating cancer can be precious. *Fusobacterium nucleatum* (*F. nucleatum*) is one of the important bacteria in the occurrence and spread of CRC. In this study, we investigated the expression levels of miR-21, miR-17-5P, miR-155, and the relative frequency of *F. nucleatum* in biopsy samples from patients with CRC.

**Method:**

DNA and RNA samples were extracted using a tissue extraction kit, and then cDNAs were synthesized using a related kit. Based on the sequence of miR-17-5P, miR-21, and miR*-*155 genes, *F. nucleatum* specific 16srRNA and bacterial universal16srRNA specific primers were selected, and the expression levels of the target genes were analyzed using the Real-Time PCR method.

**Results:**

The expression level of miR-21, miR-17-5P, and miR-155 genes showed a significant increase in the cancer group. Also, the expression of the mentioned miRNAs was significantly raised in the positive samples for *F. nucleatum* presence. The relative frequency of *F. nucleatum* in the cancer group was significantly increased compared to the control group.

**Conclusion:**

Due to the changes in the expression of genes involved in causing CRC in the presence of *F. nucleatum*, it is possible to prompt identification and provide therapeutic solutions to cancer patients by studying their microbial profiles and the expression changes of different selected genes.

## Introduction

Colorectal cancer (CRC) is one of the most common malignancies among men and women [1]. CRC is the second-deadliest cancer and the third leading cause of death worldwide According to 2020 reports [2], this cancer affects about 1.9 million people annually [3]; CRC is therefore one of the fatal cancers, so early detection and treatment are critical [4]. Early stages of CRC are often asymptomatic, making on-time diagnosis an important clinical challenge. Due to the multifactorial nature of cancer, and since microbes, especially bacteria, can be among the important factors, investigating the mechanism of bacterial carcinogenesis or their role in cancer progression is crucial [5, 6]. There has been evidence that certain human cancers can be related to some microorganisms [7]. Metagenomic evaluations of intestinal microbiota show an innumerable phylogenetic diversity with an estimated more than 1000 phylotypes in the human population, with at least 160 species common to each individual [8, 9]. Changes in intestinal microbiota are associated with a variety of diseases, including CRC. The CRC-associated intestinal microbiota is rich in opportunistic proinflammatory pathogens such as Fusobacterium [10]. Various studies have shown an increase in Fusobacterium species in patients with CRC compared with healthy individuals and inflammatory tissue of the colon in the precancerous phase [11, 12]. *Fusobacterium nucleatum* (*F. nucleatum*) is an anaerobic gram-negative bacterium that uses its pathogenic factors, such as adhesion molecules and lipopolysaccharide, to induce inflammation and activate the immune system's immune responses through various cellular signaling pathways [13, 14]. According to the results of studies on the early detection and diagnosis of CRC, various genetic factors can be used as molecular markers for the early detection of CRC, including the family of miRNAs [15]. miRNAs are small non-coding regions in RNA that play an important role in regulating all biological pathways in living organisms [16]. Under normal physiological conditions, these factors can be involved in various cellular functions such as proliferation, differentiation, and apoptosis. miRNAs can also be associated with various human diseases [17, 18]. There is a lot of evidence that shows a significant increase and decrease in the levels of miRNAs in common human cancers [19], including the rise in miR-21 in CRC [20], which can affect the metabolism of tumor cells, stimulate angiogenesis, reduce regulation of tumor inhibitory genes, promote escape from immune monitoring, and create favorable conditions for tumor invasion [21, 22]. Increased miR-21 expression is also involved in metastasis [20]. In addition, miR-17-5P is highly expressed in people with CRC, miR-17-5P expression is altered in the metastatic and miR-17-5P has been proposed as a marker for the detection of CRC metastasis stage [23]. Alteration in miR-155 expression also plays a role in tumor suppression and plays a role in inducing the WNT/β-catenin pathway, miR-155 expression inhibits metastasis at the stage when adenoma-carcinoma enters the metastatic stage [24]. On the other hand, the presence of bacteria such as *F. nucleatum* may affect the expression level of the mentioned genes. To determine whether the presence of this bacterium affects the expression of genes that are involved in CRC development, in this study, the expression of miR-21, miR-17-5P, and miR-155 genes and the relative presence of *F. nucleatum* in biopsy specimens of patients with CRC and healthy individuals were investigated by Real-Time PCR.

## Method

### Sampling

In the study, 20 samples were taken from healthy people with suspected CRC who had colonoscopies, and 40 samples were taken from the colon and rectum of patients with CRC as a cancer group. Colonoscopy biopsies were obtained from the right (from the cecum to transverse) and left (from descending to the rectum) colons of patients. Tissue biopsies were collected in Transystem tubes containing normal saline and RNA-later. Tissues were stored in a freezer (− 20 °C) for analysis.

### DNA and RNA extraction and cDNA synthesis

DNA and RNA extraction from biopsy specimens was performed using a DNA extraction kit and RNA extraction for tissue samples (Rojeh Company- Iran). A spectrophotometer (NanoDrop, 2000) was used to measure the concentration and purity of the extracted DNA. The synthesis of cDNAs was performed using a cDNA synthesis kit (RT-Roset, Rojeh Company- Iran).

### Real-time PCR

Real-Time PCR was performed using the specific primers mentioned in Table 1 to evaluate the expression changes of the selected genes and the relative abundance of *F. nucleatum*. Quantitative PCR reactions were performed on Real-Time PCR Applied Biosystems 7900 using SYBR^®^ select Master Mix in 20 μl reactions. Cycle conditions for the miR-21, miR-17-5P, and miR-155 genes were as follows: 95 °C for 5 min (activation of Taq DNA polymerase) and 40 cycles at 95 °C for 30 s, 60 °C for 30 s and 72 °C for 30 s. Cycle conditions for the detection of *F. nucleatum* were as follows: 95 °C for 5 min (activation of Taq DNA polymerase), and 40 cycles at 95 °C for 20 s, 56 °C for 30 s, and 72 °C for 20 s.

**Table 1:** Primers used in this study

Gene	Primer sequences 5′-3′	References
*gapdh*	F: ATGTTGTGCCTACCTCCATCT	[24]
	R: GGTGCTAAGCAGTTGGTGGT	
miR-155	F: ACACTCCAGCTTAATGCTAATCGTGATAG	[25]
	R: CTCAACTGGTGTCGTGGA	
miR-21	F: GCCCGCTAGCTTATCAGACTGATG	[26]
	R: CAGTGCAGGGTCCGAGGT	
miR-17-5P	F: ACACTCCAGCTGGGCAAAGTGCTTACAGTGCA	[27]
	R: TGGTGTCGTGGAGTCGGC	
*16srRNA*- *F. nucleatum*	F: GATCCAGCAATTCTGTGTG	[28]
	R: CGAATTTCACCTCTACACTTG	
*16srRNA*-Universal	F: AGMGTTYGATYMTGGCTCAG	[29]
	R: GCTGCCTCCCGTAGGAGT	

## Reference gene qPCR

The *gapdh* cellular gene was used to normalize the target genes expression in biopsy specimens for miRNA genes and the bacterial universal 16srRNA gene was used as a reference gene to evaluate the relative abundance of *F. nucleatum* (Table 1). All qPCR reactions for controls and tests were evaluated in duplicate.

### Statistical analysis

Biopsy specimens from the cancer group (n = 40) and control group (n = 20) in terms of presence and relative frequency of *F. nucleatum,* as well as relative expression of miR-21, miR-17-5P*,* and miR-155 genes, were analyzed.

The formula 2^−ΔΔCt^ was used to determine the relative expression of each miRNA to *gapdh* RNA.

$$\Delta \Delta {\text{Ct }} = \, \Delta {\text{Ct }}\left( {{\text{Target}}} \right){-}\Delta {\text{Ct }}\left( {{\text{Reference}}} \right)$$

Fold change of target genes expression was calculated using the following formula.

$${2}^{{ - \left( {{\text{Ct Target }} - {\text{ Ct Reference}}} \right){\text{ Tumor}}}} {/2}^{{ - \left( {{\text{Ct Target }} - {\text{ Ct Reference}}} \right){\text{ Normal}}}}$$

SPSS version 21 and PRISM software version 8 were used for data analysis. Quantitative data were summarized as mean and reaction progression deviation. Quantitative data were examined for normal distribution, and in the case of normal distribution, analysis of variance (Non-parametric ANOVA) with a significant level (*P* value > 0.05) was used.

## Results

### Samples

**Figure1:**
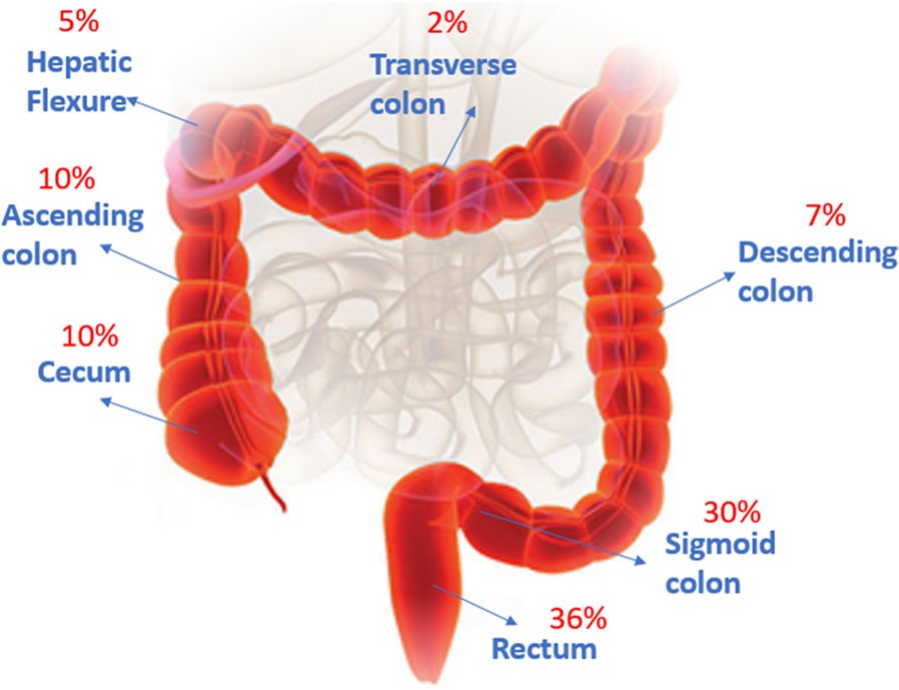
Types of cancer samples examined in this study

In this study, the subjects in the cancer group included 52% women and 48% men, with the age range between 50–60 and 50–80 years in the women and men groups, respectively. The subjects in the control group included 45% women and 55% men, among whom the highest age range was between 30–40 and 30–50 years for women and men, respectively. Among the evaluated patients, the most common symptoms that led to colonoscopy were anemia (34%), abdominal pain (31%), blood in the stool (19%), and rectal bleeding (16%). Figure 1 shows the frequency of different gut parts’ involvement in CRC based on the gastroenterologist's initial examinations and the pathologists’ microscopic examinations. The morphological diversity of tissue samples in this study included adenocarcinoma (87%) and adenoma (13%). The tissue samples studied include the Proximal and Distal sections of intestinal tissue. A complete description of cancer samples is shown in Table 2.

**Table 2:** Pathological information of patients with CRC

Patients			Tumor		
Sample ID	Age	Sex	Location	Size (cm)	Morphology
C01	42	F	Ascending colon	0.3 × 0.2 × 0.1	Adenocarcinoma
C02	59	M	Hepatic flexure	0.5 × 0.4 × 0.2	Adenocarcinoma
C03	72	M	Rectum	1.5 × 1 × 0.3	Adenocarcinoma
C04	82	F	Sigmoid colon	1.5 × 1 × 0.7	Adenocarcinoma
C05	69	F	Sigmoid colon	0.5 × 0.4 × 0.2	Adenocarcinoma
C06	51	M	Descending colon	0.3 × 0.2 × 0.1	Adenocarcinoma
C07	49	M	Sigmoid colon	0.3 × 0.2 × 0.1	Adenocarcinoma
C08	78	M	Cecum	0.3 × 0.2 × 0.1	Adenocarcinoma
C09	68	M	Cecum	0.5 × 0.3 × 0.2	Adenocarcinoma
C10	48	F	Rectum	0.8 × 0.6 × 0.2	Adenoma
C11	76	F	Cecum	1 × 1 × 0.3	Adenocarcinoma
C12	27	M	Ascending colon	0.6 × 0.4 × 0.2	Adenocarcinoma
C13	51	F	Rectum	1 × 0.7 × 0.3	Adenocarcinoma
C14	84	M	Rectum	0.3 × 0.2 × 0.1	Adenoma
C15	70	F	Rectum	0.3 × 0.2 × 0.1	Adenocarcinoma
C16	76	F	Hepatic flexure	0.3 × 0.2 × 0.1	Adenocarcinoma
C17	56	F	Sigmoid colon	0.5 × 0.3 × 0.2	Adenocarcinoma
C18	65	M	Ascending colon	0.5 × 0.3 × 0.2	Adenocarcinoma
C19	51	M	Sigmoid colon	1 × 0.7 × 0.3	Adenocarcinoma
C20	49	M	Sigmoid colon	0.3 × 0.2 × 0.1	Adenocarcinoma
C21	63	F	Sigmoid colon	1 × 0.8 × 0.2	Adenoma
C22	58	M	Sigmoid colon	0.9 × 0.7 × 0.3	Adenocarcinoma
C23	64	M	Descending colon	0.3 × 0.2 × 0.1	Adenocarcinoma
C24	52	M	Rectum	1 × 0.9 × 0.2	Adenocarcinoma
C25	58	F	Ascending colon	0.6 × 0.2 × 0.2	Adenoma
C26	45	M	Descending colon	0.7 × 0.5 × 0.2	Adenocarcinoma
C27	56	F	Rectum	0.3 × 0.2 × 0.1	Adenocarcinoma
C28	86	M	Rectum	0.3 × 0.2 × 0.1	High grade glandular dysplasia
C29	73	M	Rectum	0.3 × 0.2 × 0.1	Adenocarcinoma
C30	59	F	Rectum	0.3 × 0.2 × 0.1	Adenocarcinoma
C31	63	F	Rectum	0.3 × 0.2 × 0.1	Adenocarcinoma
C32	73	M	Cecum	1 × 0.5 × 0.5	Adenocarcinoma
C33	57	M	Sigmoid colon	0.7 × 0.6 × 0.1	Adenocarcinoma
C34	58	F	Sigmoid colon	0.6 × 0.5 × 0.2	Adenocarcinoma
C35	71	F	Rectum	1.5 × 1 × 0.2	Adenoma
C36	62	M	Transverse colon	0.8 × 0.5 × 0.2	Adenocarcinoma
C37	78	M	Rectum	0.3 × 0.2 × 0.1	Adenocarcinoma
C38	78	F	Sigmoid colon	0.3 × 0.2 × 0.1	Adenocarcinoma
C39	53	F	Sigmoid colon	0.3 × 0.2 × 0.1	Adenocarcinoma
C40	66	F	Rectum	0.3 × 0.2 × 0.1	Adenocarcinoma

*F* Female, *M* Male

### The expression level of target genes

**Fig. 2:**
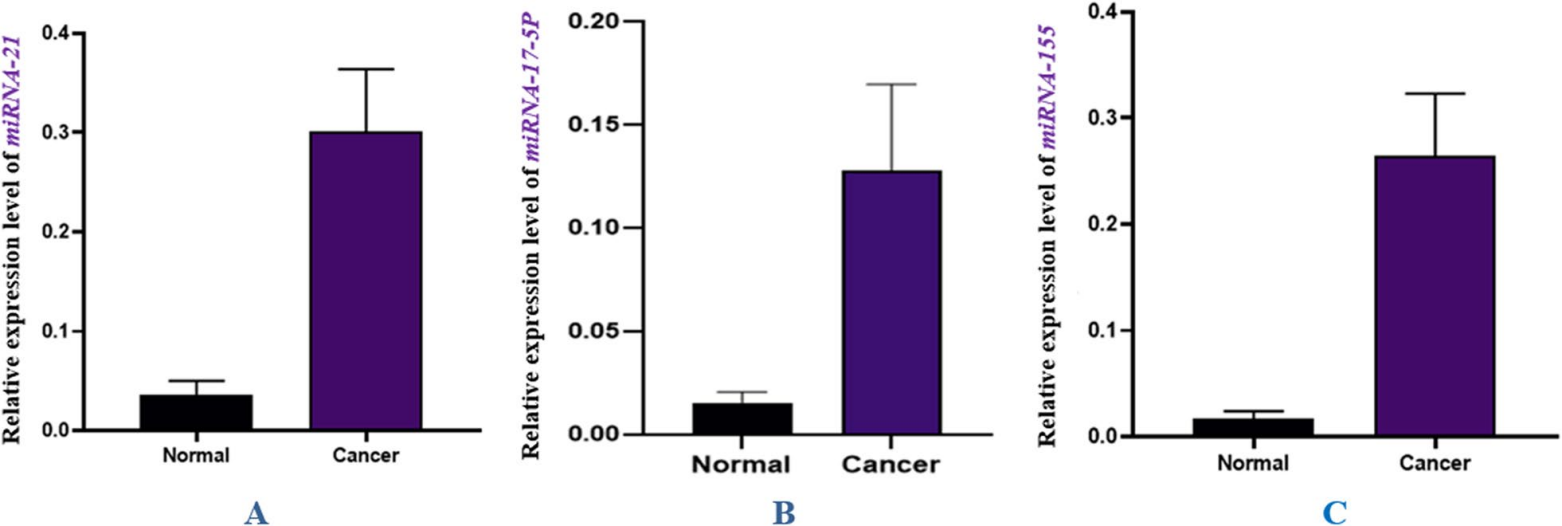
Comparison of the expression level of **A** miR-21*,* **B** miR-17-5P, and miR-155 genes in cancer and control groups

**Fig. 3:**
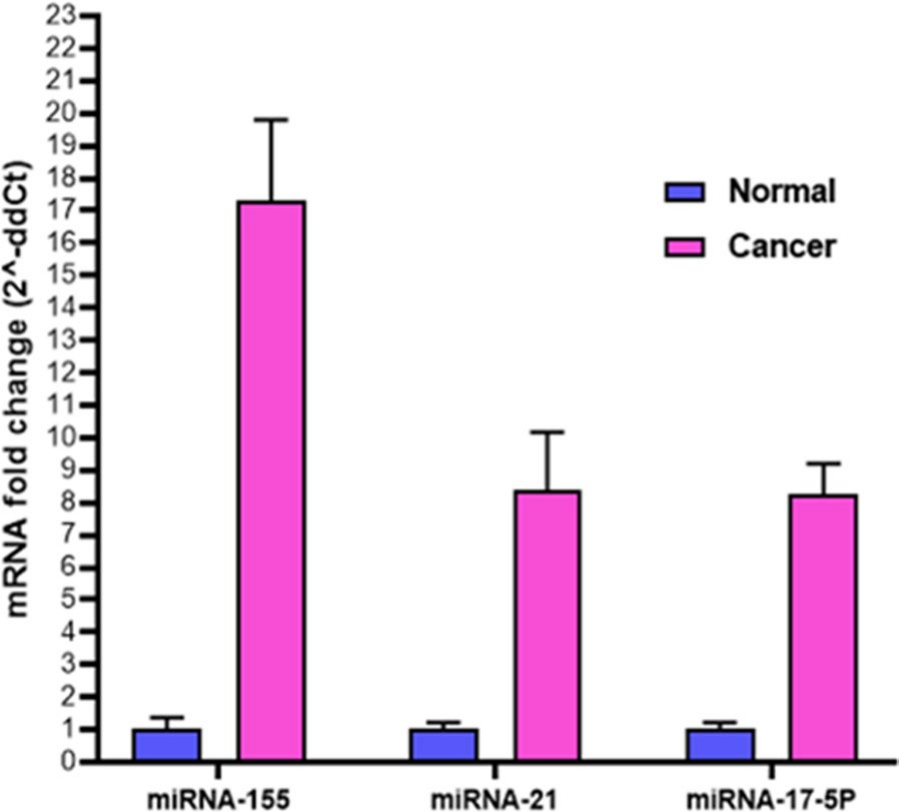
Fold change analysis of miR-21, miR-17-5P, and miR-155 genes expression in the cancer group relative to the control cancer groups

**Fig. 4:**
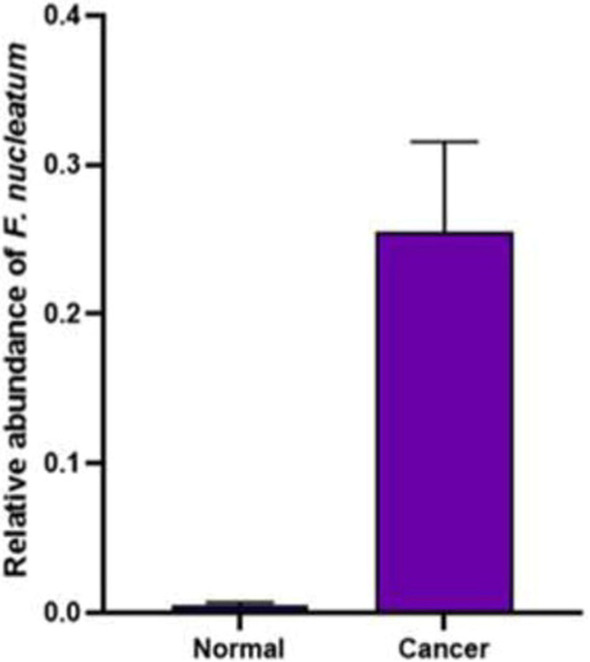
Comparison of the presence of *F. nucleatum- 16srRNA* gene in cancer and control groups

As a control, the *gapdh* gene was used to evaluate the expression of miR-21, miR-17-5P, and miR-155. Real-Time PCR was used to estimate *gapdh* gene expression levels in cancer and control group samples. According to the results, a comparison of miR-21 gene expression in control and cancerous groups shows that the miR-21 gene in the cancer group significantly increased compared to the control group (*P* value = 0.0058). Comparison of miR-17-5P expression in control and cancer groups showed that the miR-17-5P gene significantly increased in cancer compared to the control group (*P* value = 0.0194). A comparison of miR-155 expression in healthy and cancerous groups indicated that this gene’s expression significantly increased in the cancer group compared to the control (*P* value = 0.0005). A comparison of the expression of these genes in the two groups is shown in Fig. 2. As determined by fold change Analysis of miRNAs genes, the level of miR-21 gene expression was 17 times higher in the cancer group than in the control group (*P* value = 0.005), while miR-17-5P and miR-155 gene expression increased by over eight times in comparison to the control group (*P* value = 0.019) (Fig. 3). In order to estimate the relative abundance of the bacterium, the 16srRNA gene primers specific for *F. nucleatum* were used. The results indicated that the frequency of *F. nucleatum* in the cancer group was significantly higher than in the control group (*P* value = 0.0243) (Fig. 4).

### *F. nucleatum* abundance in cancer and control groups

For all samples taken from two control groups and the cancer group, real-time PCR was performed using 16 s rRNA specific for *F. nucleatum*. The relative frequency of *F. nucleatum* in the cancer group was significantly higher than in the control. The relative frequency of *F. nucleatum* was 70% in the cancer group and 25% in the control group. In addition, the results of the present study showed that *F. nucleatum* was more prevalent in men than in women with cancer since 58% of men and 42% of women were positive for the bacterium presence. There was also a difference in the relative prevalence of *F. nucleatum* among cancer patients in different age groups, with a greater prevalence among men between 50 and 80 and women between 70 and 80.

### Relative abundance of *F. nucleatum* in different types of CRC samples

**Fig. 5:**
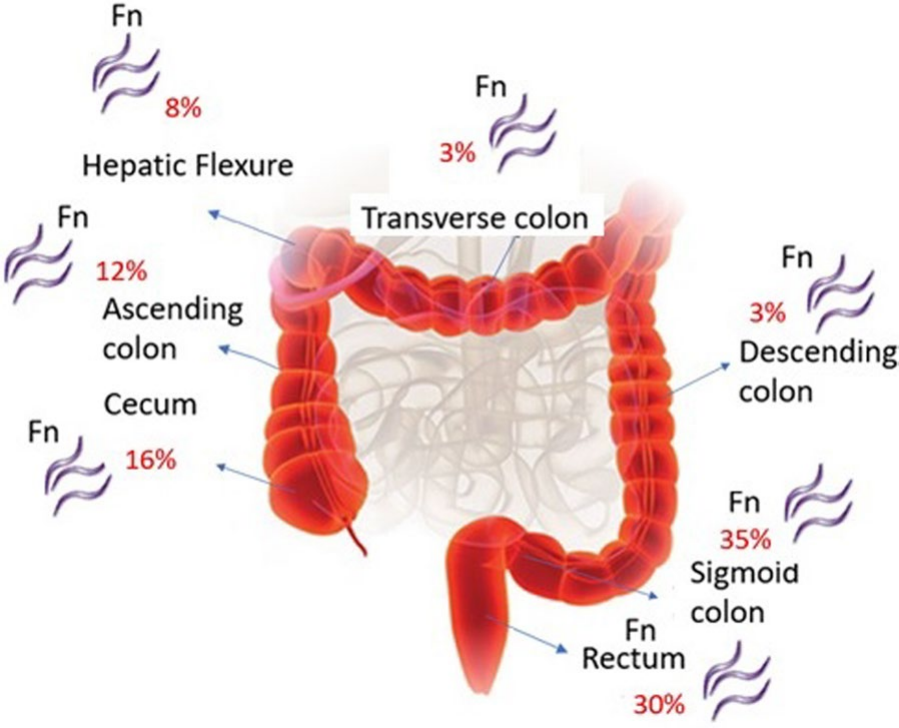
Relative abundance of *F. nucleatum* in cancer samples taken from the different parts of the colon

Investigations of the association between tumor position and frequency of *F. nucleatum* showed that *F. nucleatum* is present in 63% of tumors in the distal part of the colon and 27% of tumors in the proximal part of the colon and rectum. According to our results, tumors located in the distal part of the colon were more frequently associated with *F. nucleatum* (Fig. 5). Results indicated that *F. nucleatum* was more prevalent in cancer specimens with adenocarcinoma morphology than in other morphologic types. *F. nucleatum* was detected in 71% of cancer specimens with adenocarcinoma morphology and 13% with adenoma morphology. According to the results, the relative distribution of *F. nucleatum* in different parts of the large intestine is different; the most abundance was observed in the sigmoid, rectum, cecum, ascending colon, hepatic flexion, descending colon, and transverse colon, respectively (Fig. 5).

### Changes in the relative expression levels of the selected genes in the presence and absence of *F. nucleatum*

**Fig. 6:**
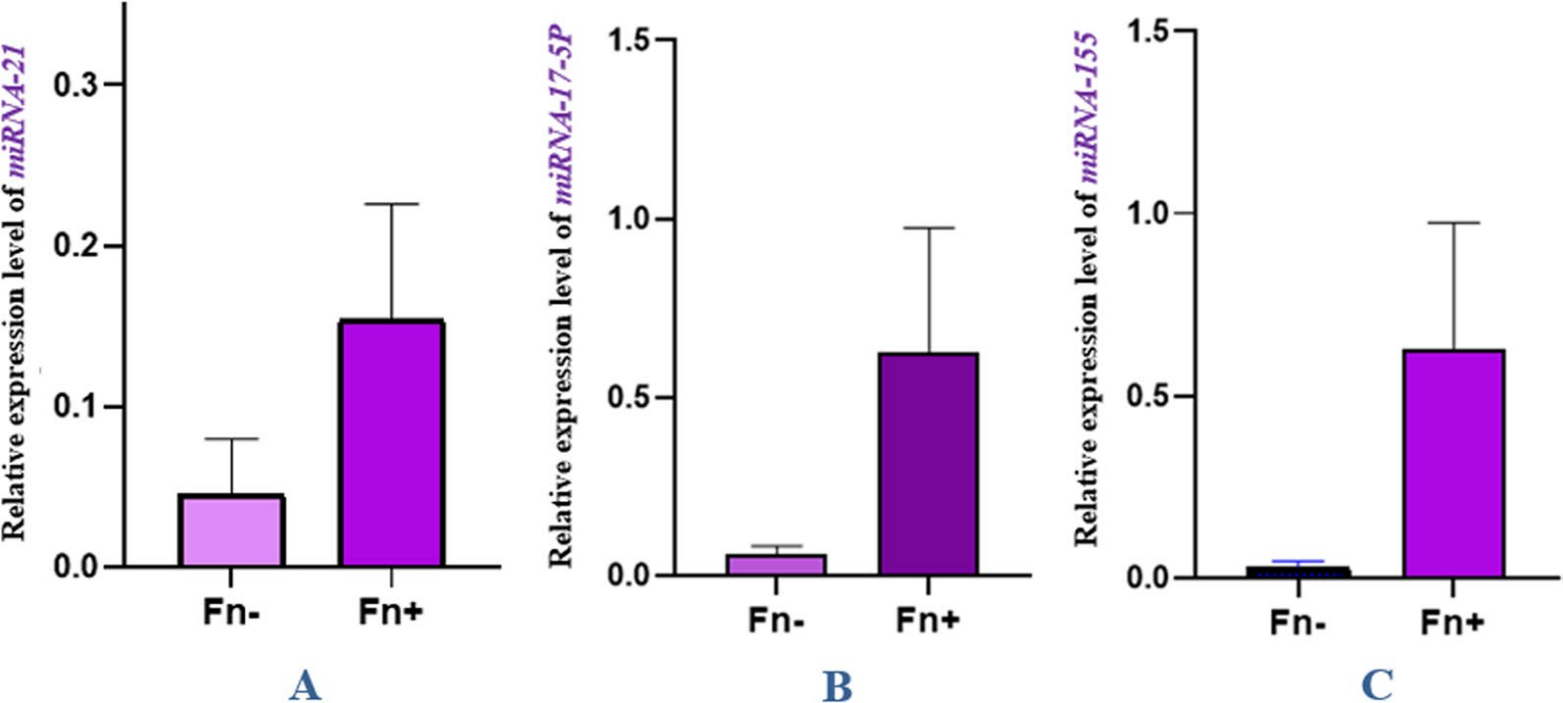
Comparison of the relative presence of *F. nucleatum- 16srRNA* and the expression level of **A** miR-21*,* **B** miR-17-5P, and **C** miR-155 genes in cancer samples

Based on a comparison of miR-21, miR-17-5P, and miR-155 expression levels in cancer group samples with and without *F. nucleatum,* it was found that *F. nucleatum*-positive cancer specimens displayed a higher increase in these genes’ expression relative to *F. nucleatum*-negative cancer specimens. In order to assess the level of expression of miR-21, miR-17-5P, and miR-155 genes, a fold change analysis was performed. According to the results, miR-21 and miR-17-5P expression levels were increased by about 3.5 and 8 times in the *F. nucleatum*-positive cancer group compared to the *F. nucleatum*-negative cancer group, respectively. In addition, miR-155 expression levels increased about 17 times in the *F. nucleatum*-positive cancer group (Fig. 6).

## Discussion

Colorectal cancer (CRC) is one of the most common cancers worldwide; host–pathogen interactions may play an essential role in development of some types of cancers. In the current study, the expression of miR-21, miR-17-5P, and miR-155 genes in presence and absence of *F. nucleatum* in biopsy specimens of patients with CRC and healthy individuals were evaluated. In the present study, different parts of the right and left colon were examined, including the sigmoid (30%), rectum (36%), cecum (10%), ascending colon (10%), descending colon (7%), hepatic flexion (5%), transverse colon (2%), the results of the current study also showed a higher commonness of CRC in the left part of the colon than in the right part, especially in the sigmoid and rectum. A study by Komiya et al. in Japan (2018) examined biopsy specimens of the ascending colon (46%), sigmoid (30%), and rectum (24%) of patients with CRC. This study found a higher incidence of CRC in the ascending colon, contrary to our study, their results indicate that CRC occurs more frequently in the rectum [30]. In another study by Tunsjo et al. in Norway (2019), various biopsy specimens were examined from different parts of the colon, including the sigmoid (44%), cecum (24%), ascending colon (13%), rectum (13%), and transverse colon (8%). Unlike the current study, the hepatic flexion section was not examined in their study nevertheless, overall results were similar to ours [31]. In light of the importance of CRC, studying its causes is crucial. It is possible to provide effective prevention and treatment solutions by understanding the causes of these diseases. CRC, like other cancers, can start with mutations in specific genes, such as miRNAs that play an essential role in several intracellular signaling mechanisms [32]. Changing miRNA genes' expression disrupts these genes' function, which can induce cancer [33]. The current study found a significant increase in the miR-21, miR-17-5, and miR-155 genes’ expression in patients with CRC. Evaluation of miRNA gene expression levels using real-time PCR in our study revealed that miR-21 gene expression significantly increased times in the cancer group compared to the healthy group. On the other hand, the miR-17-5P and miR-155 gene expression was also raised. According to a study by Shibuya et al. in Japan (2011), the expression of miR-21 and miR-155 genes in the cancer group increased more than 7 and 10 times, respectively; the expression of these genes [34]. Also, according to the findings of the study by Nassar and his colleagues in Lebanon (2021), using serum samples of CRC and healthy individuals, the expression of miR-21 and miR-155 genes in the cancer group was increased by more than 4 and 3 times, respectively [35]. In addition to colorectal cancer, the change in miR-21 gene expression is particularly important in other cancers, such as lung, breast, and stomach cancer [36–38]. The same as our study, other studies, including Huang et al. in China [39], Ibrahiem et al. in Saudi Arabia (2020) [40], and Sun et al. in China [41] the expression level of miR-17-5P gene in CRC patients showed a significant increase. It has been shown that CRC patients’ intestinal microbiota is dominated by opportunistic proinflammatory pathogens such as Fusobacterium. CRC outcomes are influenced by *F. nucleatum*. TLR4 and MYD88 signals are activated in the presence of this bacterium, and miR-21 expression is upregulated*, NF-B* is activated, and *RASA1* is suppressed, increasing CRC cell proliferation [20, 25, 26]. In addition, the presence of *F. nucleatum* was also associated with poor prognoses in CRC patients and probably contributed to chemoresistance, therefore, if it is determined that the frequency of this bacterium is higher in cancer tissues, it can be considered a potential marker for predicting the development or occurrence of CRC [27, 28]. Comparing CRC and control tissue samples, we found that people with CRC have a significantly higher amount of *F. nucleatum* presence than those without cancer. In this study, the presence of *F. nucleatum* was seen in 70% and 25% of samples taken from cancer and control groups, respectively. As in the present study, Shariati and colleagues in Iran conducted a study comparing biopsy samples of control and CRC patients in 2020 and found that the relative frequency of *F. nucleatum* was higher in CRC patients compared to controls; 23% of cancer patients and 13% of controls exhibited *F. nucleatum* [29]. An increase in the relative abundance of *F. nucleatum* was also reported in the study by Tunsjo and her colleagues in Norway (2019) conducted on stool samples from people with CRC and healthy people [31]. The present study evaluated the relative expression of miR-21, miR-17-5P, and miR-155 related to the presence and absence of *F. nucleatum* in the cancer group samples. Compared with the absence of *F. nucleatem,* the selected genes displayed higher expression in the presence of this bacterium. These results can partially confirm the effect of this bacteria in causing cancer by changing the expression level of some miRNAs. In general, most studies conducted in Iran and other countries in the field of CRC investigated the expression changes of important miRNAs, especially miR-21, miR-17-5P, and miR-155, regardless of the influence of microbial factors such as *F. nucleatum.* The present study is noteworthy since it has evaluated the expression changes of these genes independently and in the presence of *F. nucleatum* in CRC patients.

## Conclusion

The results of the present study show an increase in the expression of specific miRNA genes in cancer samples compared to the control. We also found that the relative frequency of the presence of bacteria increased in CRC biopsy samples compared to healthy individuals. It was also found that the expression of studied miRNAs in *F. nucleatum*-positive cancer samples is significantly higher compared to cancer samples without the detectable level of presence of this bacterium. All these results show the importance of investigating the presence of this bacterium in CRC-confirmed or suspected samples in order to prognosis cancer progression more quickly and also to prevent its development. Also, considering the important role of miRNAs and their increase in cancer samples, with further studies, they can be considered as biomarkers for the possible detection of the presence of cancer or its advanced level.

## Data Availability

The original data source could be shared upon the request of the principal investigator.

## References

[1] Center MM, Jemal A, Ward E (2009). International trends in colorectal cancer incidence rates. Cancer Epidemiol Biomark Prev.

[2] Xi Y, Xu P (2021). Global colorectal cancer burden in 2020 and projections to 2040. Trans Oncol.

[3] Jemal A, Bray F, Center MM, Ferlay J, Ward E, Forman D (2011). Global cancer statistics. CA: Cancer J Clin.

[4] Siegel EM, Jacobsen PB, Lee J-H, Malafa M, Fulp W, Fletcher M (2014). Florida initiative for quality cancer care: improvements on colorectal cancer quality of care indicators during a 3-year interval. J Am Coll Surg.

[5] Liu W, Zhang X, Xu H, Li S, Lau HC-H, Chen Q (2021). Microbial community heterogeneity within colorectal neoplasia and its correlation with colorectal carcinogenesis. Gastroenterology.

[6] Anghel SA, Ioniță-Mîndrican C-B, Luca I, Pop AL (2021). Promising epigenetic biomarkers for the early detection of colorectal cancer: a systematic review. Cancers.

[7] Sepich-Poore GD, Zitvogel L, Straussman R, Hasty J, Wargo JA, Knight R (2021). The microbiome and human cancer. Science.

[8] Chu Y, Sun S, Huang Y, Gao Q, Xie X, Wang P (2021). Metagenomic analysis revealed the potential role of gut microbiome in gout. npj Biofilms Microb.

[9] Tripathi VP, Goo D, Maidya BN, Aneebuddin M (2022). New trends in interrelation of infectious colorectal cancer with intestinal microbiota. Arch Gastroenterol Res.

[10] Li R, Shen J, Xu Y (2022). Fusobacterium nucleatum and colorectal cancer. Infect Drug Resist.

[11] Mira-Pascual L, Cabrera-Rubio R, Ocon S, Costales P, Parra A, Suarez A (2015). Microbial mucosal colonic shifts associated with the development of colorectal cancer reveal the presence of different bacterial and archaeal biomarkers. J Gastroenterol.

[12] Slade DJ (2021). New roles for *Fusobacterium*
*nucleatum* in cancer: target the bacteria, host, or both?. Trends Cancer.

[13] Gur C, Ibrahim Y, Isaacson B, Yamin R, Abed J, Gamliel M (2015). Binding of the Fap2 protein of *Fusobacterium*
*nucleatum* to human inhibitory receptor TIGIT protects tumors from immune cell attack. Immunity.

[14] Wu Z, Ma Q, Guo Y, You F (2022). The role of *Fusobacterium*
*nucleatum* in colorectal cancer cell proliferation and migration. Cancers.

[15] Ghafouri-Fard S, Hussen BM, Badrlou E, Abak A, Taheri M (2021). MicroRNAs as important contributors in the pathogenesis of colorectal cancer. Biomed Pharmacother.

[16] Hussen BM, Hidayat HJ, Salihi A, Sabir DK, Taheri M, Ghafouri-Fard S (2021). MicroRNA: a signature for cancer progression. Biomed Pharmacother.

[17] Hsieh P, Yamane K (2008). DNA mismatch repair: molecular mechanism, cancer, and ageing. Mech Ageing Dev.

[18] Uzuner E, Ulu GT, Gürler SB, Baran Y. The role of MiRNA in cancer: pathogenesis, diagnosis, and treatment. miRNomics: Springer; 2022. p. 375–422. 10.1007/978-1-0716-1170-8_18 34432288

[19] Bitaraf A, Razmara E, Bakhshinejad B, Yousefi H, Vatanmakanian M, Garshasbi M (2021). The oncogenic and tumor suppressive roles of RNA-binding proteins in human cancers. J Cell Physiol.

[20] Liu T, Liu D, Guan S, Dong M (2021). Diagnostic role of circulating MiR-21 in colorectal cancer: a update meta-analysis. Ann Med.

[21] Niu L, Yang W, Duan L, Wang X, Li Y, Xu C (2021). Biological implications and clinical potential of metastasis-related miRNA in colorectal cancer. Mol Ther-Nucleic Acids.

[22] Zhang W, Jiang Z, Tang D (2022). The value of exosome-derived noncoding RNAs in colorectal cancer proliferation, metastasis, and clinical applications. Clin Transl Oncol.

[23] Fu F, Jiang W, Zhou L, Chen Z (2018). Circulating exosomal miR-17-5p and miR-92a-3p predict pathologic stage and grade of colorectal cancer. Transl Oncol.

[24] Gao Y, Han T, Han C, Sun H, Yang X, Zhang D (2021). Propofol regulates the TLR4/NF-κB pathway through miRNA-155 to protect colorectal cancer intestinal barrier. Inflammation.

[25] Zhang Y, Zhang L, Zheng S, Li M, Xu C, Jia D (2022). Fusobacterium nucleatum promotes colorectal cancer cells adhesion to endothelial cells and facilitates extravasation and metastasis by inducing ALPK1/NF-κB/ICAM1 axis. Gut Microb.

[26] Bakshi HA, Quinn GA, Nasef MM, Mishra V, Aljabali AA, El-Tanani M (2022). Crocin inhibits angiogenesis and metastasis in colon cancer via TNF-α/NF-kB/VEGF pathways. Cells.

[27] Liu Y, Baba Y, Ishimoto T, Tsutsuki H, Zhang T, Nomoto D (2021). Fusobacterium nucleatum confers chemoresistance by modulating autophagy in oesophageal squamous cell carcinoma. Br J Cancer.

[28] Hong J, Guo F, Lu S-Y, Shen C, Ma D, Zhang X (2021). *F*. *nucleatum* targets lncRNA ENO1-IT1 to promote glycolysis and oncogenesis in colorectal cancer. Gut.

[29] Shariati A, Razavi S, Ghaznavi-Rad E, Jahanbin B, Akbari A, Norzaee S (2021). Association between colorectal cancer and *Fusobacterium*
*nucleatum* and Bacteroides fragilis bacteria in Iranian patients: a preliminary study. Infect Agents Cancer.

[30] Komiya Y, Shimomura Y, Higurashi T, Sugi Y, Arimoto J, Umezawa S (2019). Patients with colorectal cancer have identical strains of *Fusobacterium*
*nucleatum* in their colorectal cancer and oral cavity. Gut.

[31] Tunsjø HS, Gundersen G, Rangnes F, Noone JC, Endres A, Bemanian V (2019). Detection of *Fusobacterium*
*nucleatum* in stool and colonic tissues from Norwegian colorectal cancer patients. Eur J Clin Microbiol Infect Dis.

[32] Huang X, Zhu X, Yu Y, Zhu W, Jin L, Zhang X (2021). Dissecting miRNA signature in colorectal cancer progression and metastasis. Cancer Lett.

[33] Dragomir MP, Knutsen E, Calin GA (2021). Classical and noncanonical functions of miRNAs in cancers. Trends Genet.

[34] Shibuya H, Iinuma H, Shimada R, Horiuchi A, Watanabe T (2010). Clinicopathological and prognostic value of microRNA-21 and microRNA-155 in colorectal cancer. Oncology.

[35] Nassar FJ, Msheik ZS, Itani MM, Helou RE, Hadla R, Kreidieh F (2021). Circulating mirna as biomarkers for colorectal cancer diagnosis and liver metastasis. Diagnostics.

[36] Koopaie M, Abedinejad F, Manifar S, Mousavi R, Kolahdooz S, Shamshiri A (2021). Salivary miRNA-21 expression as a potential non-invasive diagnostic biomarker in breast cancer. Gene Rep.

[37] Alexandre D, Teixeira B, Rico A, Valente S, Craveiro A, Baptista PV (2022). Molecular beacon for detection miRNA-21 as a biomarker of lung cancer. Int J Mol Sci.

[38] Ma S, Kong S, Gu X, Xu Y, Tao M, Shen L (2021). As a biomarker for gastric cancer, circPTPN22 regulates the progression of gastric cancer through the EMT pathway. Cancer Cell Int.

[39] Huang C, Liu J, Xu L, Hu W, Wang J, Wang M (2019). MicroRNA-17 promotes cell proliferation and migration in human colorectal cancer by downregulating SIK1. Cancer Manage Res.

[40] Ibrahiem AT, Fawzy MS, Abu AlSel BT, Toraih EA (2021). Prognostic value of BRAF/MIR-17 signature and B-Raf protein expression in patients with colorectal cancer: a pilot study. J Clin Lab Anal.

[41] Sun W, Cui J, Ge Y, Wang J, Yu Y, Han B (2022). Tumor stem cell-derived exosomal microRNA-17-5p inhibits anti-tumor immunity in colorectal cancer via targeting SPOP and overexpressing PD-L1. Cell Death Discov.

